# High Reflectance Nanoscale V/Sc Multilayer for Soft X-ray Water Window Region

**DOI:** 10.1038/s41598-017-13222-5

**Published:** 2017-10-10

**Authors:** Qiushi Huang, Qiang Yi, Zhaodong Cao, Runze Qi, Rolf A. Loch, Philippe Jonnard, Meiyi Wu, Angelo Giglia, Wenbin Li, Eric Louis, Fred Bijkerk, Zhong Zhang, Zhanshan Wang

**Affiliations:** 1 Key Laboratory of Advanced Micro-Structured Materials MOE, Institute of Precision Optical Engineering, School of Physics Science and Engineering, Tongji University, Shanghai, 200092 China; 2 Institute of Nuclear Physics and Chemistry, China Academy of Engineering Physics, Mianyang, 621900 China; 3 Shanghai Institute of Laser Plasma, China Academy of Engineering Physics, Shanghai, China; 4 The Max Planck Institute for the Structure and Dynamics of Matter, Center for Free Electron Laser Science, LuruperChaussee 149, Hamburg, 22761 Germany; 5 Sorbonne Universités, UPMC Univ Paris 06, Laboratoire de Chimie Physique-Matière et Rayonnement, 11 Rue Pierre et Marie Curie, F-75231 Paris Cedex 05, Paris, France; 6 CNRS UMR 7614, Laboratoire de Chimie Physique-Matière et Rayonnement, 11 Rue Pierre et Marie Curie, F-75231 Paris Cedex 05, Paris, France; 7 CNR Istituto Officina Materiali, 34149 Trieste, Italy; 8 Industrial Focus Group XUV Optics, MESA+ Institute for Nanotechnology, University of Twente, P.O. Box 217 7500 AE, Enschede, The Netherlands

## Abstract

V/Sc multilayer is experimentally demonstrated for the first time as a high reflectance mirror for the soft X-ray water window region. It primarily works at above the Sc-L edge (λ = 3.11 nm) under near normal incidence while a second peak appears at above the V-L edge (λ = 2.42 nm) under grazing incidence. The V/Sc multilayer fabricated with a d-spacing of 1.59 nm and 30 bilayers has a smaller interface width (σ = 0.27 and 0.32 nm) than the conventional used Cr/Sc (σ = 0.28 and 0.47 nm). For V/Sc multilayer with 30 bilayers, the introduction of B_4_C barrier layers has little improvement on the interface structure. As the number of bilayers increasing to 400, the growth morphology and microstructure of the V/Sc layers evolves with slightly increased crystallization. Nevertheless, the surface roughness remains to be 0.25 nm. A maximum soft X-ray reflectance of 18.4% is measured at λ = 3.129 nm at 9° off-normal incidence using the 400-bilayers V/Sc multilayer. According to the fitted model, an s-polarization reflectance of 5.2% can also be expected at λ = 2.425 nm under 40° incidence. Based on the promising experimental results, further improvement of the reflectance can be achieved by using a more stable deposition system, exploring different interface engineering methods and so on.

## Introduction

X-ray microscopy particularly working in the water window region (λ = 2.3–4.4 nm) provides a powerful method for imaging biological samples *in vitro* due to the natural optical contrast between water and carbon^1^. It is an important complimentary technique to the visible light and electron microscopy. The water window X-ray microscope has been widely developed based on the synchrotron radiation sources^2,3^ and lab-based X-ray sources^4^, using either the classic imaging optical system^2,4^ or the new diffraction imaging technique^3,5^. The X-ray free electron lasers (FEL) have also generated ultrashort pulses with extremely high brilliance in this range, which can image the biological structure with a single pulse and avoid the radiation damages^6,7^.

**Figure 1:**
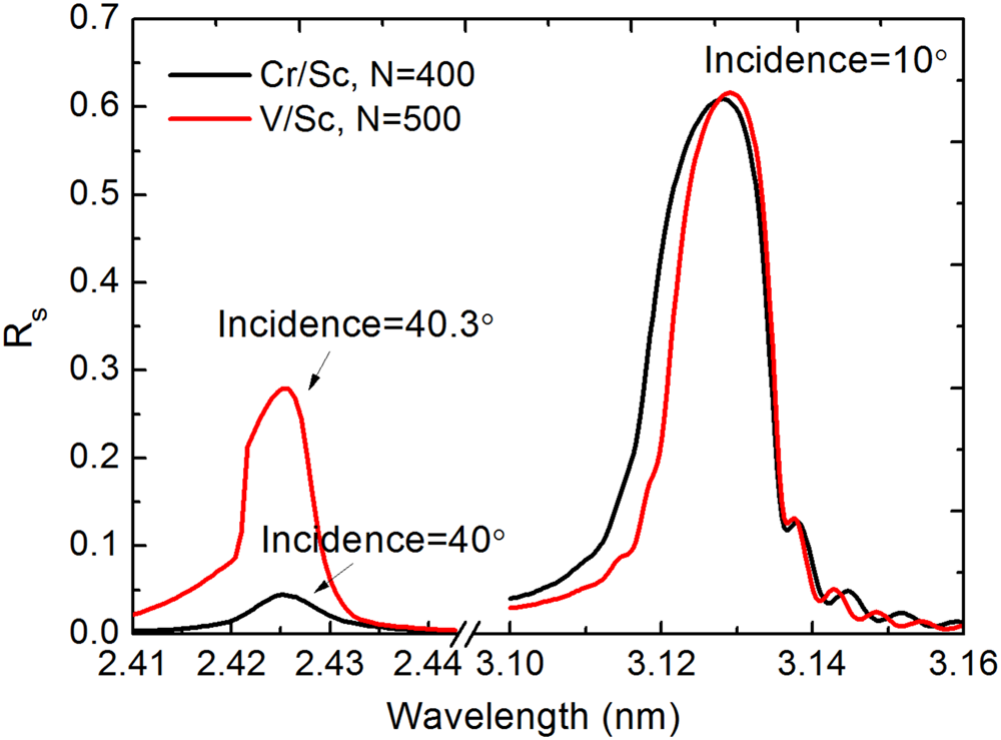
Theoretical s-polarized reflectance curves of V/Sc and Cr/Sc multilayers. No interface width is added in the structure. Both multilayers have a d-spacing of 1.59 nm and Γ = 0.5.

In these applications, X-ray optics are the crucial components to focus the photons to the sample and transport the scattered ones to the detector. Multilayer mirror is one of the optics that has long been developed as the collector or objective lens for the microscope^4,8^. It has also been used to introduce a time-delay for the pump-probe imaging experiment^7^. Fabrication of the multilayers for the water window region is a challenge due to the extremely small layer thickness of less than 1 nm. Thus, atomic scale imperfections at the layer interfaces, including roughness and inter-diffusion, can degrade the experimental reflectance dramatically. To improve the interface structure, interface engineering methods of introducing barrier layers^9–11^, reactive sputtering with nitrogen^12^, or ion assistance^13^ have been studied. These methods were applied on the conventional multilayer candidates, like Cr/Sc, Cr/Ti, and Cr/V and a highest reflectance in the water window region of 32% was achieved with Cr/Sc working at above the Sc-L edge (λ = 3.11 nm)^9^. Despite the experimental progress, the measured reflectance is still far below the theoretical value, which is 60% at around λ = 3.13 nm for Cr/Sc (Fig. 1). For a collector mirror used in real application, the reflectance can be even lower^4^.

Therefore, to further enhance the achievable reflectance, new materials can be explored. V/Sc was recently proposed to work near the Sc-L edge by Loch *et al*.^14^, based on the larger positive enthalpy of formation between Sc and V as compared to Sc and Cr^15^. This leads to a lower miscibility between the material pairs, i.e., low inter-diffusion and low chemical reactivity, which could manifest itself in sharper interfaces and, hence, a higher experimental reflectance despite the lower optical contrast. The maximum theoretical reflectance of the V/Sc multilayer with 1.59 nm d-spacing and the thickness ratio of Sc to the d-spacing as Γ = 0.5 is similar to that of Cr/Sc, as shown in Fig. 1 (the optical constants are compiled from the atomic scattering factors from the Center for X-ray Optics (CXRO) and the Lawrence Livermore National Laboratory (LLNL) in the IMD software^16^). The optical contrast between V and Sc is lower than Cr and Sc, which brings a larger number of saturated bilayers and smaller bandwidth. Moreover, the same V/Sc multilayer can also work near the V-L edge (λ = 2.42 nm) with close to 30% reflectance (s-polarization) if the incidence angle changes from near normal incidence to grazing incidence as shown in Fig. 1. This is a unique advantage compared to the Cr/Sc multilayer. Furthermore, the theoretically predicted single-shot damage threshold of V/Sc under the irradiation of FEL is also a little higher than Cr/Sc, which provides the possibility of using it in FEL facilities^14^. In order to explore these proposed advantages, we recently fabricated and characterized a series of V/Sc multilayers and a high soft X-ray (SXR) performance is demonstrated.

## Results

### Structural characterization of the deposited multilayers

The V/Sc multilayer was designed and fabricated with a d-spacing of 1.59 nm and Γ = 0.5 for working at near normal incidence. The number of bilayers is N = 30. A Cr/Sc multilayer with the same structure parameters was also fabricated as a comparison. The B_4_C capping layer with 2–3 nm thickness was added on the top of all multilayers to prevent oxidation. This capping layer will only induce about 1% drop (absolutely) of the theoretical reflectance of the multilayer. The multilayers were deposited on super-polished silicon wafers with a root-mean-square (RMS) roughness of 0.25 nm.

**Figure 2:**
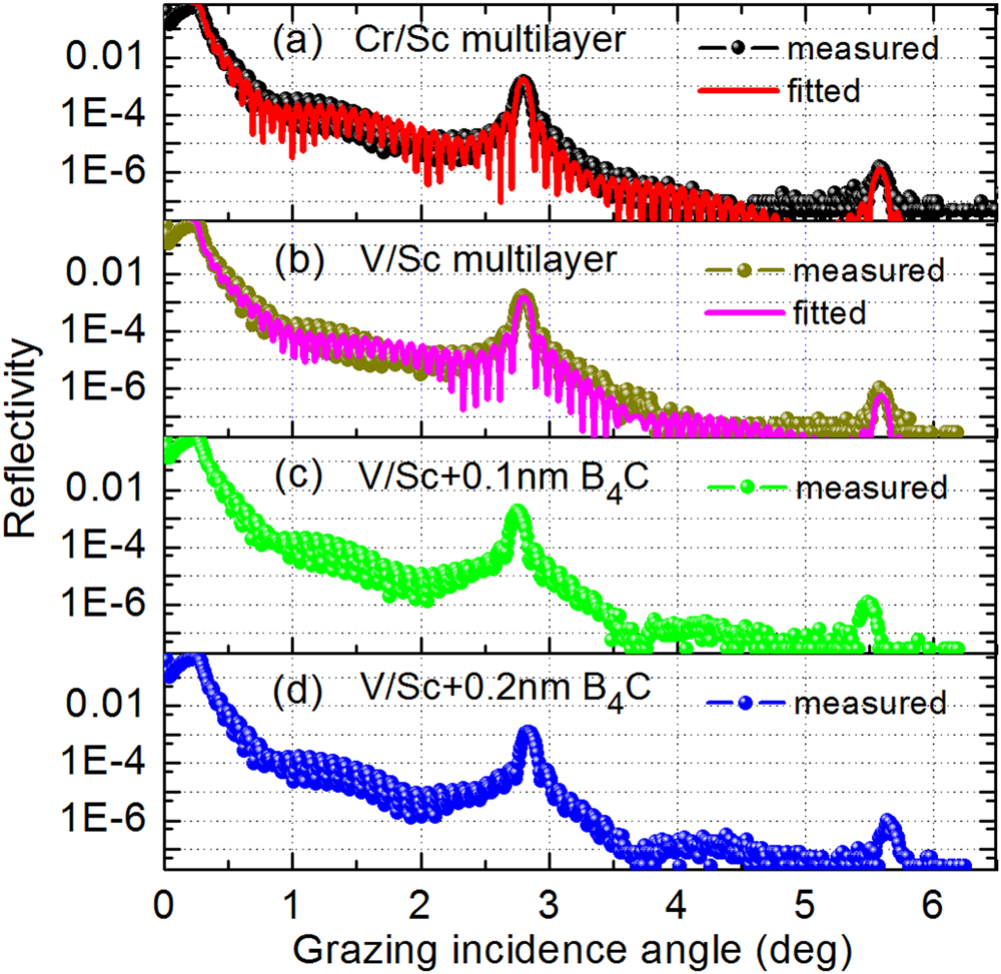
Grazing incidence x-ray reflectance measurements of 30 bilayers Cr/Sc multilayer **(a)**, V/Sc multilayer **(b)**, V/Sc with 0.1 nm **(c)** and 0.2 nm thickness B_4_C **(d)** at both interfaces. The fitted results of pure Cr/Sc and V/Sc multilayers are also shown in **(a)** and **(b)**.

**Figure 3:**
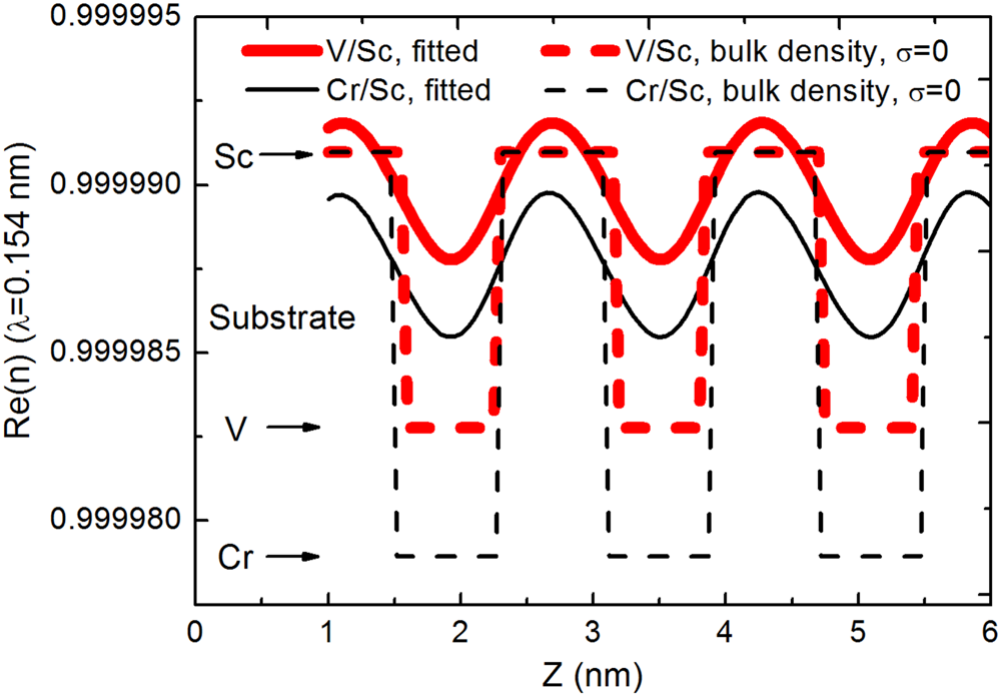
Real part of the refractive index profile of the V/Sc (thick lines) and Cr/Sc (thin lines) multilayers with ideal layer structure (dashed lines) and experimentally fabricated structure (solid lines).

The multilayers were characterized by grazing incidence X-ray reflectometry (GIXR) at Cu-Kα emission line (λ = 0.154 nm) and the results are shown in Fig. 2. The GIXR curves of the Cr/Sc and V/Sc multilayers were fitted using the IMD software to determine the interface widths^16^. Here, the interface width represents the effects from both interface roughness and diffusion. The fitted results of the Cr/Sc multilayer (Fig. 2(a)) exhibits relatively large interface widths of σ = 0.28 nm and 0.47 nm for the two interfaces which seems to be asymmetric. This asymmetry has been confirmed by Haase *et al*. based on complementary measurements where the interface width of Cr-on-Sc is larger^17^. For the V/Sc multilayer (Fig. 2(b)), the fitted interface widths are σ = 0.27 nm and 0.32 nm for the two interfaces. Although the GIXR fitting result alone cannot provide the exact asymmetry information of the two interfaces, the average interface width of V/Sc is significantly smaller than Cr/Sc. Based on the GIXR fitted models, the refractive index profiles (real part) of the layer structures were calculated and shown in Fig. 3. The index profile of the ideal structure is also shown as a comparison. Although the ideal Cr/Sc multilayer has a higher optical contrast than V/Sc, the poor interface structure in reality significantly dilutes the contrast and the deviation between the theoretical and experimental profiles is much larger than the case of V/Sc. It is evident that the pure V/Sc multilayer displays sharper interfaces than Cr/Sc, which can provide higher SXR reflectance in reality. This result is consistent with the expectation that a slightly larger positive enthalpy of formation and the corresponding lower miscibility between V and Sc, as compared to Cr and Sc, results in smaller interface widths after deposition.

In order to further study the effect of barrier layers on the V/Sc multilayer, two samples with B_4_C barriers of 0.1 nm and 0.2 nm thickness were fabricated, respectively. The B4C “layers” were added at both interfaces. This barrier layer was proved to be very effective in reducing the interface width of Cr/Sc^9^ and Cr/V multilayers^11,18^. For the two samples, the V and Sc layer thickness were decreased equally in order to keep the d-spacing of 1.59 nm unchanged. Due to the unknown chemical state of the boron and carbon atoms of the ultrathin barrier “layers”, the V/B_4_C/Sc/B_4_C multilayers were not fitted. For the multilayer with 0.1 nm B_4_C barrier layers (Fig. 2(c)), the reflectance of the 1st order peak is 0.18% that is similar to the one of pure V/Sc (0.16% in Fig. 2(b)), taken into account the slightly different peak position. With 0.2 nm B_4_C barrier layers (Fig. 2(d)), the 1st order peak reflectance drops to 0.12%. Nevertheless, the d-spacing is somewhat reduced that can explain the smaller reflectance. It is indicated that the B_4_C barrier layers have little effect on the structure quality of the V/Sc multilayer with 30 bilayers, which is noticeably different from the case of the Cr/Sc and Cr/V multilayers. Therefore, in the following discussions, the V/Sc multilayers are all fabricated with no barrier layer.

**Figure 4:**
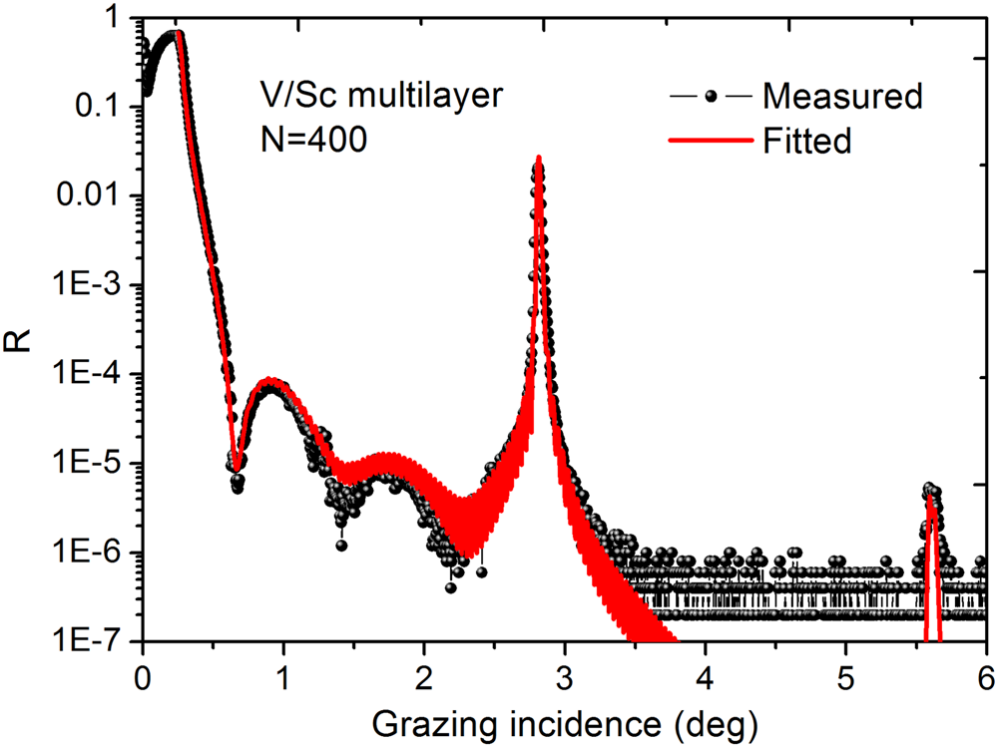
Grazing incidence x-ray reflectance measurement and the fitted curve of the 400-bilayers V/Sc multilayer.

To reach the maximum reflectance of an ideal V/Sc multilayer at near normal incidence, a bilayer number of 500 is needed. If the interface widths are taken into account in the structure, the saturated number of bilayers increases to ~700. Be that as it may, given the finite long term stability of the deposition facility, a V/Sc multilayer with 400 bilayers was fabricated for demonstration purposes. The GIXR measurement and the fitted results are shown in Fig. 4. The instrumental angular width of 0.007° and the layer thickness drift were taken into account in the fitting model. The whole stack were assumed to have the same interface widths for each period which were fitted as σ = 0.29 nm and 0.35 nm, for the two interfaces. A small d-spacing decrease of ~0.020 nm from bottom to top of the stack was found which can be attributed to the systematic drift of the deposition rate during the long time sputtering. The interface widths of the 400-bilayers multilayer are slightly larger than the one with 30 bilayers. This result will be further confirmed by the fitted model of the SXR reflectivity and the reason will be discussed in the following part.

**Figure 5:**
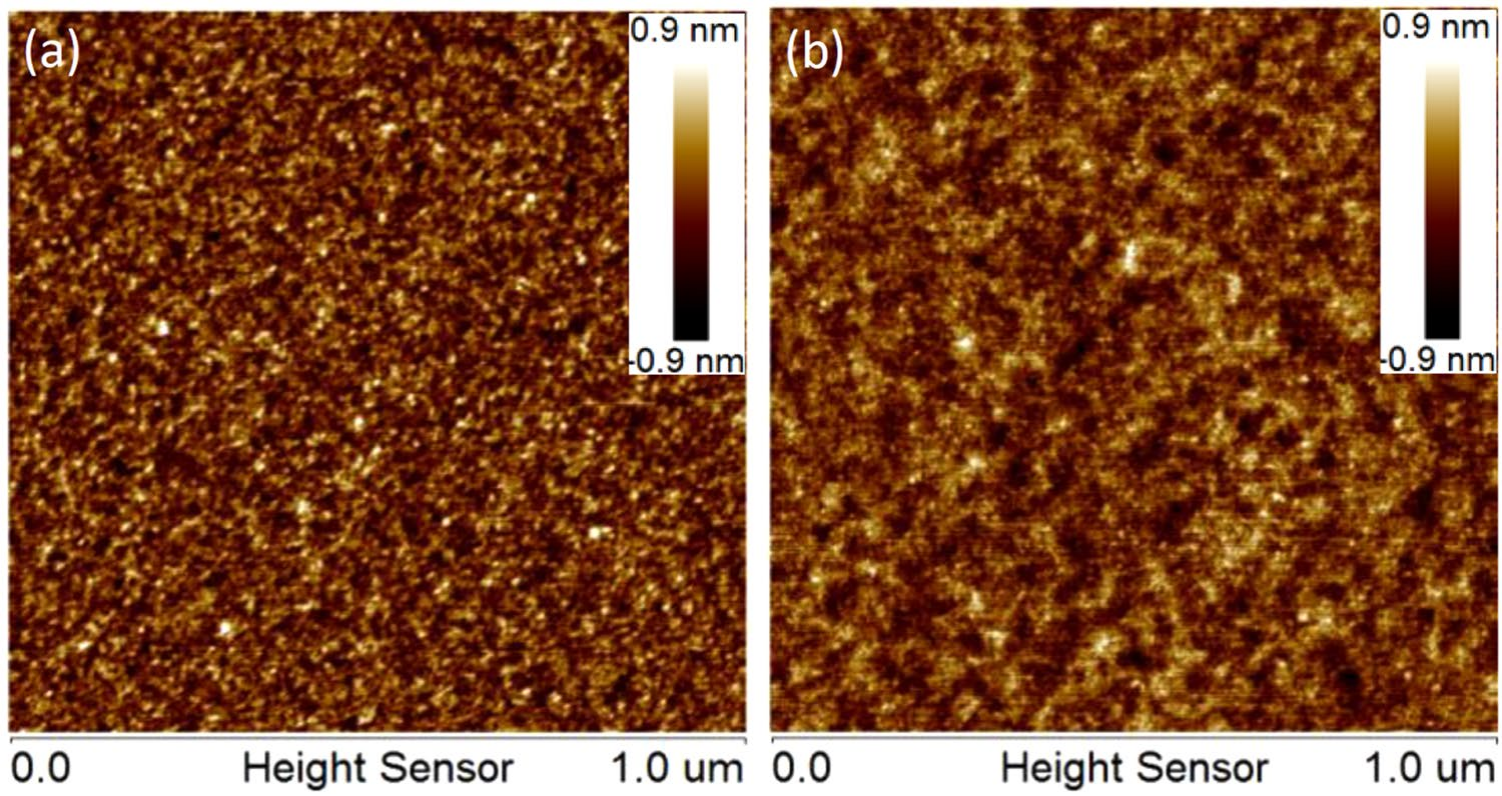
AFM images of the surface morphology of V/Sc multilayers with 30 bilayers (**a**) and 400 bilayers (**b**).

The surface morphology of the multilayers with 30 and 400 bilayers were measured by AFM. Figure 5 shows the images taken over an area of 1 × 1 μm^2^ with 512 × 512 points. Both samples exhibit a very smooth surface with an RMS roughness of only 0.24–0.25 nm, which is the same as the super polished Si substrates. However, the fine structure of the surface morphology is different between the two samples. The typical feature size on the surface of the 400-bilayers sample is larger than that of the 30-bilayers sample. According to the two dimensional power spectral density analysis of the two surfaces, the roughness component with relatively low spatial frequency (5 μm^−1^ > f > 21 μm^−1^) is slightly increased after the growth of 400 bilayers, while the high frequency component (21 μm^−1^ < f < 130 μm^−1^) is suppressed. It implies that the layer growth morphology changes with the deposition of 400 bilayers

**Figure 6:**
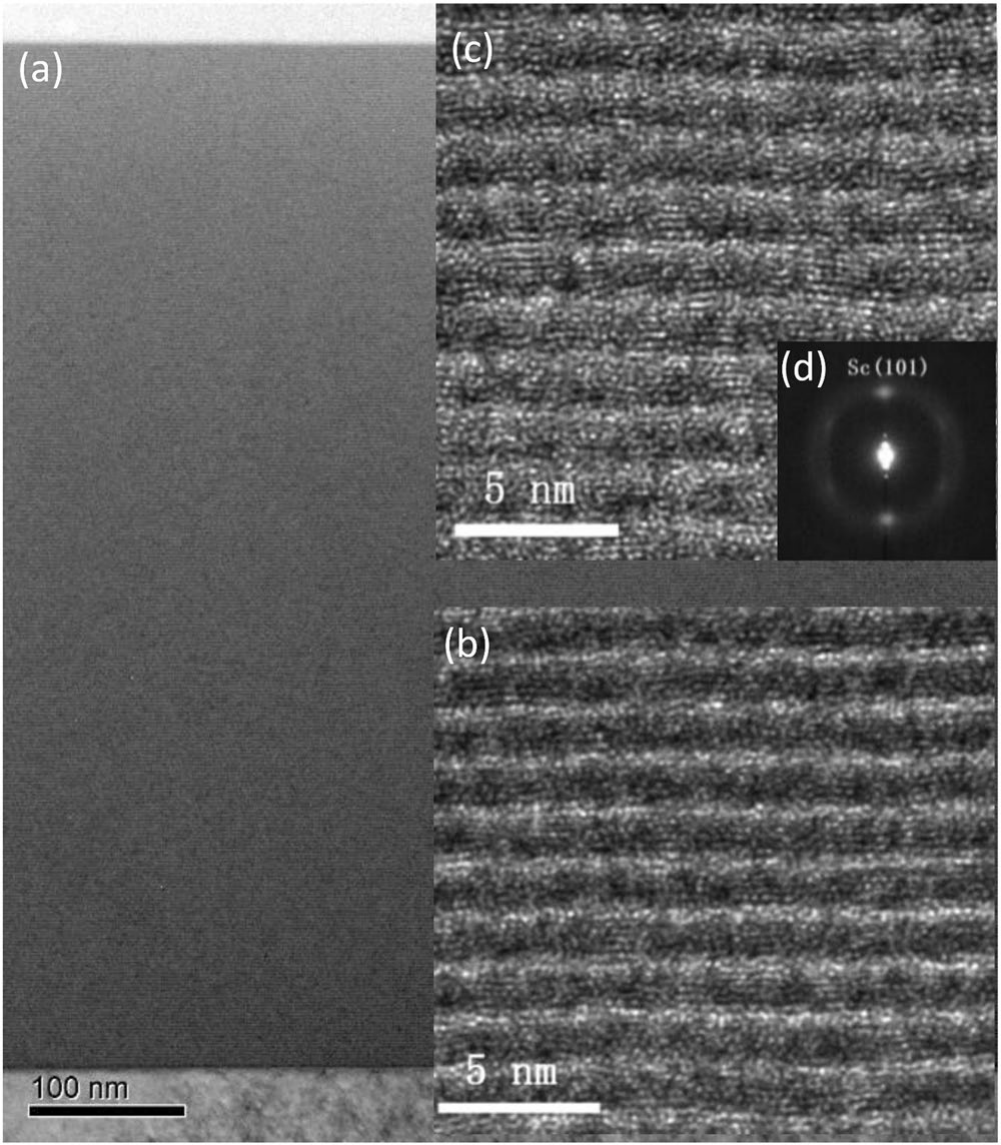
TEM images of the whole 400-bilayers V/Sc multilayer stack **(a)**, the bottom layers **(b)**, middle layers **(c)**, and the SAED pattern of the middle layers **(d)**. Sc is depicted by the bright layers and V by the dark layers.

To further study the internal layer structure of the multilayer, the sample with 400 bilayers was measured with transmission electron microscopy (TEM). High resolution images were taken at the bottom area of the stack (close to substrate), at the middle area, and at the top. The results are shown in Fig. 6, where the bright layers are Sc and the dark layers are V. The first layers grown at the bottom display mostly amorphous structure, only very few tiny grains can be observed (Fig. 6(b)). However, at the middle area, the crystallization is enhanced with more ordered arrangement of atoms and the grains are randomly distributed inside the layers. The layer structure at the top area is similar to the middle part and is not shown in the figure. The selected area electron diffraction pattern (SAED) was further measured at the middle part of the stack where the diffraction spot is identified as hexagonal Sc (101). No obvious diffraction pattern of vanadium is observed, suggesting that it remains amorphous. The microstructure transition from the almost amorphous state to the polycrystalline state with the increase of bilayers was also reported in the Ni/V multilayer^13^. The polycrystalline growth of the layers can increase the low frequency roughness at the interfaces, which explains the evolved surface morphology of the 400 bilayers as compared to 30 bilayers (Fig. 5). On the other hand, the Cr/Sc multilayers with the same d-spacing (1.59 nm) exhibit an amorphous structure for both Cr and Sc layers^13,19^ while the crystallization of Sc was only observed as the d-spacing increased to above 2.2 nm^19^. It is implied that the Sc layers start to crystallize from a smaller thickness in the V/Sc system as compared to the Cr/Sc.

To investigate possible layer impurities, energy dispersive X-ray spectroscopy (EDX) was also performed at the cross-section of the multilayer and a small amount of oxygen and nitrogen were detected in the layers. Nevertheless, the absolute atomic concentration cannot be determined due to the lack of standard reference sample in the measurement.

### Soft X-ray reflectance of the V/Sc multilayer

In order to study the above-mentioned effects on the actual optical performance, the SXR reflectivity of the multilayer with 400 bilayers was measured at the Bending magnet for Emission, Absorption, and Reflectivity (BEAR) beamline at the ELETTRA synchrotron^20^. The incident beam was set as almost 100% s-polarization, with an energy bandwidth of 0.4 eV (full width half maximum), and the wavelength was scanned around the Sc-L edge. The sample was measured at the near normal incidence of 7° and 9°, respectively, to study the peak reflectance at different wavelengths. The results are shown in Fig. 6. The measured reflectance at 3.142 nm wavelength with the incidence angle of 7° is 15.1%. With the larger incidence of 9°, the Bragg peak shifts closer to the Sc-L edge and a higher reflectance of 18.4% was obtained at λ = 3.129 nm. The measured reflectance curves were fitted and the energy bandwidth of the incident beam and the layer thickness drift over the stack are taken into account. Nevertheless, the layer impurities like oxygen, nitrogen, etc., are not included in the model as the exact amount of impurities is not determined. The fitted interface widths of the two interfaces are σ = 0.31 nm and 0.35 nm. A slight decrease of the d-spacing of around 0.017 nm is observed from the bottom to the top of the 400-bilayers stack. The SXR fitted results are very close to the GIXR results of the 400-bilayers sample (Fig. 4) which confirmed the accuracy of the fitted model. The combined analysis of GIXR and SXR measurements proves that the newly developed V/Sc multilayer has smaller interface widths than the pure Cr/Sc multilayer^17,21^. The slightly larger interface widths of the 400-bilayers multilayer as compared to the 30-bilayers one can be attributed to the increased crystallization of the layers after the growth of ~200 bilayers which may degrade the originally sharp interface. Based on the fitted results, the 0.4 eV energy bandwidth of the incident beam causes a little broadening of the Bragg peak and around 1.5% reduction (absolute value) of the peak reflectance. It means that multilayer reflectance at λ = 3.129 nm should actually be around 20% when utilizing monochromatic beam incidence. The spectral response of the multilayer near the V-L edge was not measured. Nevertheless, based on the SXR fitted model above, the reflectance profile working at the incidence of 40° is simulated. As shown in Fig. 6, a peak reflectance of 5.2% can be obtained at λ = 2.425 nm.

## Discussion

**Figure 7:**
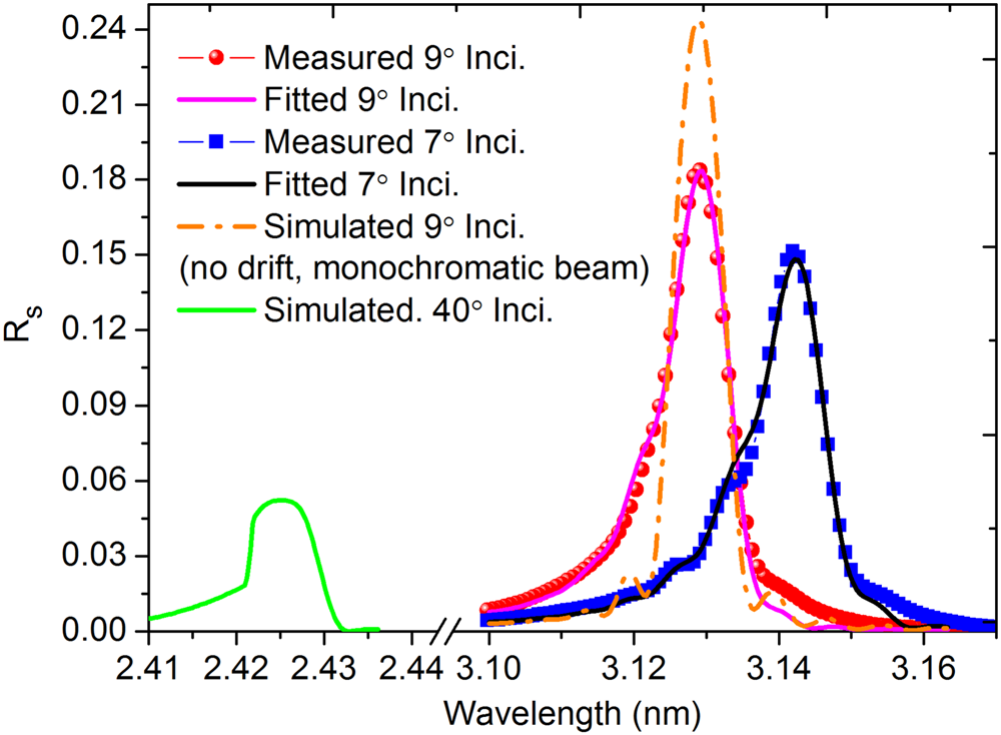
Measured SXR reflectivity curves and the fitted results of the 400-bilayers V/Sc multilayer at the incidence of 9° and 7°. The simulated reflectance curve at 40.05° incidence is also shown.

Although the measured reflectance is still lower than that of the maximum value of the highly optimized Cr/Sc multilayer (32%)^9^, our promising first attempt of the pure V/Sc multilayer does show a higher reflectance (18.4%) than the pure Cr/Sc (14.5%), while the latter even reported a factor 3 smaller thickness drift^22^. This reflectance difference can be mainly explained by a smaller interface width between V and Sc. Based on this, several methods to improve the reflectance of V/Sc can be considered. Firstly, the fabricated 400-bilayers multilayer displays a thickness drift of 17~20 pm, which can be reduced using a more stable deposition system^13^. Based on the current structure model, if the thickness drift can be fully removed and using monochromatic incident beam, a high reflectance of 24.4% can be expected with the 400 bilayers V/Sc as shown in Fig. 7. In this case, a saturated number of bilayers (N = 700) can then be deposited to reach the maximum saturated reflectance which is already 30%. Secondly, the multilayers were deposited under a moderate base vacuum of 6.0 × 10^−5^ Pa. Considering the reported chemical analysis results of the Cr/V^18^ and Cr/Sc multilayer^12^ deposited under a similar base vacuum, a small amount of impurities of O, N and C (not measured by EDX) are expected to be incorporated in the current V/Sc multilayer, probably forming oxides, carbides or nitrides. These contaminations could have a considerable effect on the reflectance, as indicated by Eriksson and thus need to be analyzed and controlled^22^. Thirdly, the V/Sc multilayer displays an increased crystallization with more bilayers that is different from the amorphous structure of Cr/Sc. This polycrystallization growth may be suppressed with the atomic scale barrier “layer” so that sharper interfaces can be expected at the middle and top part of the stack. A smoother substrate with RMS roughness smaller than 0.25 nm can also be beneficial to the formation of sharper interfaces. Moreover, other interface engineering methods like reactive sputtering with nitrogen and ion-assisted deposition technique can be explored to further enhance the reflectance. Thus, a highly efficient V/Sc multilayer mirror with the reflectance higher than the current world record can be expected which can deliver significantly more photon flux in the microscope system.

In summary, a high reflectance V/Sc multilayer mirror is demonstrated for the first time working near the Sc-L edge in the water window region. The interface widths of pure V/Sc are significantly smaller than the conventional Cr/Sc multilayer although the B_4_C barrier “layers” have little effect on the reflectance of V/Sc with 30 bilayers. A slightly increased polycrystallization of the layers is observed after the growth of 400 bilayers of the pure V/Sc structure. A maximum reflectance of 18.4% is measured at λ = 3.129 nm under near normal incidence of 9°. Further improvement of the reflectance can be expected through a more stable deposition process, accurate control of the layer impurities, and different interface engineering methods as have been investigated on the Cr/Sc system, that is currently widely used in this region. Moreover, this multilayer can also work near the V-L edge under grazing incidence, which is a unique advantage of this material combination and can provide double functionalities for the water window microscopes. The temporal stability and thermal stability of the V/Sc multilayer is under investigation and will be reported in the future.

## Methods

All multilayers were deposited using direct current magnetron sputtering technique on super-polished silicon wafers with a root-mean-square (RMS) roughness of 0.25 nm, as measured by atomic force microscope (AFM). The base pressure before deposition is around 6.0 × 10^−5^ Pa. High purity argon (99.999%) is used as the sputtering gas with a sputtering pressure of 0.13 Pa. The deposition rate of V, Sc, Cr, and B4C is 0.03 nm/s, 0.08 nm/s, 0.15 nm/s, and 0.01 nm/s, respectively. The grazing incidence hard X-ray reflectance and soft X-ray reflectance measurement results of the deposited multilayers were fitted using the IMD software. In the fitting model, the layer thicknesses, layer densities and interface widths were all set as fitting parameters. Error function was used as the interface profile function to modify the Fresnel reflection coefficient for the simulation of the reflectance^16^. The transmission electron microscopy measurements were performed using the FEI Tecnai G2 F20 equipment by Materials Analysis Technology Inc. The selected area electron diffraction and energy dispersive X-ray spectroscopy were also performed using the same equipment.
